# Global trends and hotspots of genetic research on non-syndromic cleft lip with or without cleft palate: a bibliometric and bioinformatics analysis

**DOI:** 10.3389/fgene.2026.1907333

**Published:** 2026-10-12

**Authors:** Yuxuan Shang, Mingjian Zhao, Shuangshuang Gao, Shikang Gao, Mengru Zhu

**Affiliations:** 1 Department of Plastic Surgery, The First People’s Hospital of Xiaoshan District — Xiaoshan Affiliated Hospital of Wenzhou Medical University, Hangzhou, China; 2 Department of Plastic Surgery, The First Affiliated Hospital of Dalian Medical University, Dalian, China; 3 Department of Craniomaxillofacial Surgery, Plastic Surgery Hospital and Institute, Chinese Academy of Medical Sciences, Peking Union Medical College, Beijing, China; 4 Center for Cleft Lip and Palate Treatment, Plastic Surgery Hospital and Institute, Chinese Academy of Medical Sciences, Peking Union Medical College, Beijing, China

**Keywords:** Bibliometrics, bioinformatics, genetics, hotspots, non-syndromic cleft lip with or without cleft palate

## Abstract

**Background:**

Non-syndromic cleft lip with or without cleft palate (NSCL/P) accounts for 70% of orofacial cleft cases worldwide and causes physical dysfunction and psychological burden. Prior bibliometric studies have outlined the general NSCL/P research landscape; however, a systematic overview targeting the genetic dimension, evolutionary trends since the Human Genome Project, and dual-database cross-validation remains limited. This study mapped the global bibliometric landscape of NSCL/P genetics, identified shifting research paradigms, and functionally validated candidate genes through bioinformatic analyses of environmental risk factors.

**Methods:**

Publications from January 1990 to 31 January 2026, were retrieved from the Web of Science Core Collection and analyzed using bibliometric tools, including R-Bibliometrix, VOSviewer, and CiteSpace. Subsequently, Scopus publications were searched using the same search strategy for cross-validation. Finally, toxicant-induced and NSCL/P-related genes were screened from the GeneCards database to identify overlapping targets, followed by construction of a protein–protein interaction network and Kyoto Encyclopedia of Genes and Genomes enrichment analysis to characterize potential functional pathways.

**Results:**

A total of 1,511 eligible publications were analyzed, covering 86 countries and regions, 1,766 institutions, 6,061 authors, and 383 journals, with rapid publication growth observed between 2018 and 2021. The United States led in output and citations alongside the University of Iowa and Murray, Jeffrey C., as key contributors. Keyword clustering categorized the current studies into four major categories: genetic analysis methodologies, developmental biology and phenotypes, epidemiology and environmental factors, and molecular regulatory mechanisms. Alcohol- and nicotine-induced overlapping pathogenic genes were identified with enriched pathways involved in the regulation of embryonic craniofacial development, cell proliferation, and apoptosis.

**Conclusion:**

This study clarified the publication trends, key research forces, disciplinary distributions, and hotspots in NSCL/P genetics, revealing an ongoing shift in research focus. In silico network analyses of public databases identified distinct molecular pathways mediating alcohol- and nicotine-induced developmental disorders. These findings provide reliable evidence for future mechanistic exploration of NSCL/P pathogenesis.

## 1 Introduction

Non-syndromic cleft lip with or without cleft palate (NSCL/P) is a common congenital craniofacial anomaly, accounting for approximately 70% of all documented orofacial cleft cases (Zhang et al., 2018; Robinson et al., 2024; Sander et al., 2024). Three phenotypic subtypes are clinically recognized: isolated cleft lip, isolated cleft palate, and combined cleft lip and palate (Kulesa-Mrowiecka et al., 2024). Impaired craniofacial growth and compromised oral function are frequently observed in affected individuals and are accompanied by long-term psychological burdens that undermine their overall quality of life (Silva et al., 2018). Unlike syndromic forms, NSCL/P represents a complex polygenic disorder marked by substantial genetic heterogeneity, with disease susceptibility modulated by multiple variants with minor effects (Robinson et al., 2024; Scapoli et al., 2019). Genetic susceptibility, external environmental triggers, and interactive regulatory mechanisms influence its pathogenesis (Scapoli et al., 2019; Dixon et al., 2011). Systematically cataloging this evidence is essential for discerning research paradigms and molecular pathways, thereby facilitating translational practice.

Sequencing technologies have advanced rapidly since the 1990 Human Genome Project (Ruffo et al., 2025; Lindeboom et al., 2021). High-resolution chromosomal microarray analysis and whole-genome sequencing have enabled the genome-wide screening of causal genetic variants associated with congenital disorders (Qi et al., 2024; Hu et al., 2023). Concurrent advances in bioinformatics throughout the post-genomic era have facilitated the broader application of genome-wide association studies (GWAS) in investigating the etiologies of complex diseases (Özkaraca et al., 2025; Rotroff, 2020). These genome-scale screening efforts have expanded the catalog of susceptible genomic regions associated with NSCL/P, with over 40 susceptible loci documented, and the number is increasing (Li et al., 2022). Cumulative genetic findings have deepened the understanding of NSCL/P genetic predisposition and have verified the core signaling pathways governing embryonic craniofacial development, including TGF-β, SHH, and Wnt signaling (Oliver et al., 2020; He et al., 2025). Despite the exponential growth in genetic discoveries, the bibliometric landscape of NSCL/P genetics, particularly its temporal evolution, geographic distribution, and thematic shifts, remains poorly characterized.

Earlier bibliometric investigations have outlined general research landscapes regarding NSCL/P, whereas recent multi-ancestry genome-wide association study meta-analyses have further consolidated the catalog of susceptibility loci (Zhang et al., 2019; Xu et al., 2026; Jia et al., 2024). However, existing bibliometric studies have not focused on the genetic dimension of NSCL/P research, nor have they leveraged dual-database approaches for discovery and validation. Therefore, updated bibliometric research is required to accommodate ongoing advances in genetic research in this field. In this study, we conducted bibliometric visualization and statistical analyses of published literature over the past 3 decades to map evolutionary hotspots and research shifts within NSCL/P genetic domains. Furthermore, to functionally contextualize the bibliometrically identified candidate genes and pathways, we performed downstream bioinformatics analyses to explore the molecular mechanisms underlying environmental risk factors such as alcohol and nicotine exposure in craniofacial development. The findings of this study provide evidence-based guidance for future investigations and translational strategies for treating NSCL/P.

## 2 Materials and methods

### 2.1 Databases and search strategy

This study was designed as a bibliometric analysis combined with bioinformatics analysis of public databases, resulting in a genetic knowledge map targeting NSCL/P. Bibliometric data were retrieved from the Web of Science Core Collection (WoSCC). The search strategy was constructed using a combination of free-text keywords and Boolean operators in the topic field to maximize retrieval sensitivity. The complete search strings, including all synonyms, truncation symbols, and field tags, are presented in Supplementary Table S1. Temporal screening boundaries for literature retrieval were restricted to 36 years, with data collection spanning 1 January 1990, to 31 January 2026. Publications were limited to English articles and reviews; meeting abstracts, letters, proceedings papers, editorial materials, book chapters, notes, and reprints were excluded. The relevance of the retrieved publications was manually verified. Finally, 1,511 publications were identified as topic-related documents (Figure 1).

**FIGURE 1:**
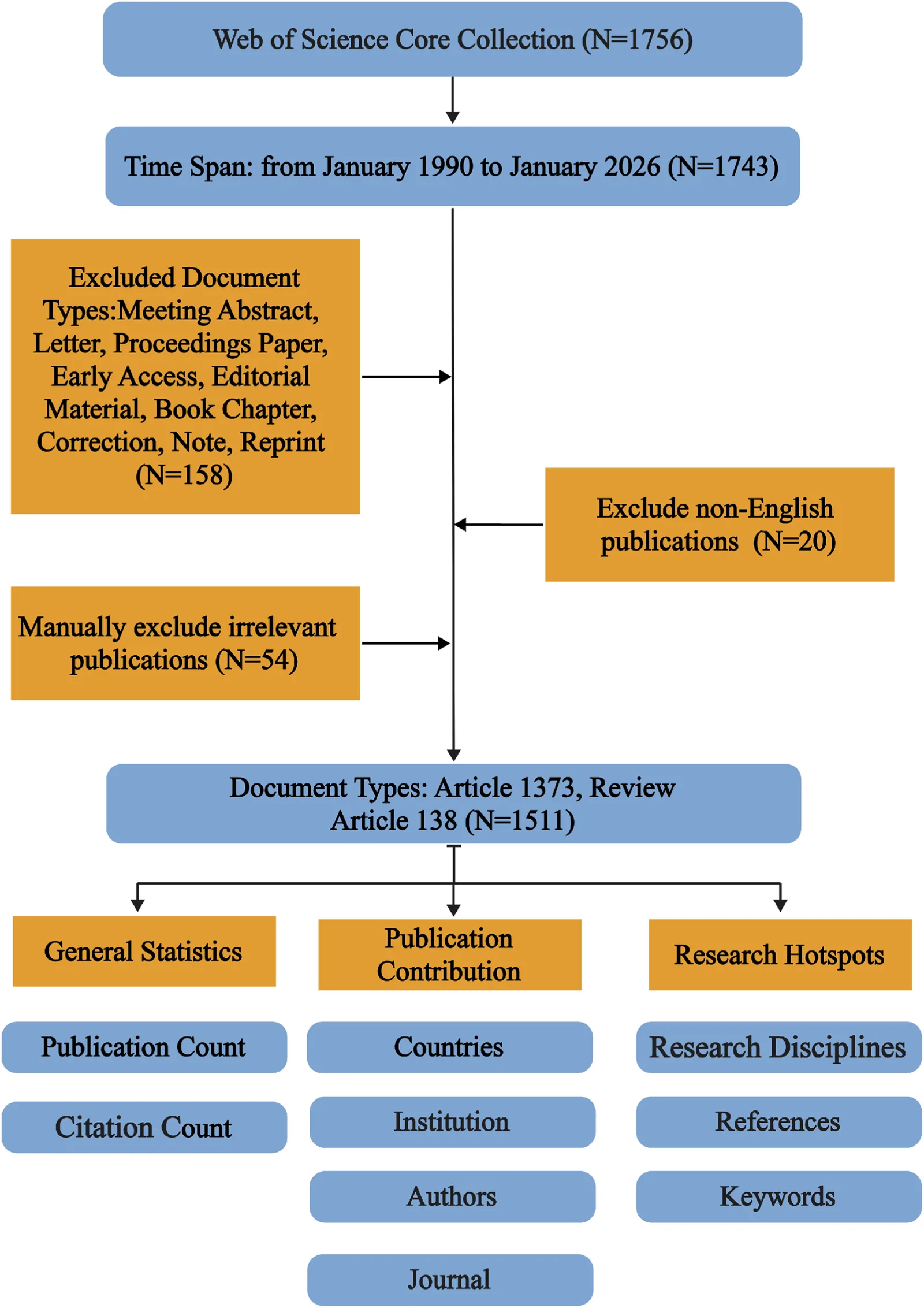
Flowchart of the search strategy and exclusion criteria for genetic studies on NSCL/P.

The Scopus database was searched using the same search strategy, document type, and language criteria to identify publications from 1 January 1990, to 31 January 2026. Publications were selected through manual screening to validate the research trends and hotspots observed in the WoSCC.

### 2.2 Data analysis and visualization

This study used Microsoft Excel 2021 to create statistical tables and trend graphs. This study employed Scimago Graphica (version 1.0.53) to analyze the countries (Hassan-Montero et al., 2022). The bibliometrix (5.0) R package in the R software (version 4.3.1) was used to conduct Lotka’s law analysis and heat mapping (Aria and Cuccurullo, 2017; Kawamura et al., 1999). Furthermore, a bibliometric analysis of the countries, institutions, authors, journals, keywords, and cited references was performed using VOSviewer (version 1.6.20) and CiteSpace (version 6.4 R1) (Chen, 2004; van Eck and Waltman, 2010). Primary attention was paid to the exploration of co-authorship, co-occurrence, and co-citation patterns. Before the data were exported for follow-up analysis, overlapping entries were merged into uniform elements, misspelled terms were manually corrected, and data were rigorously cleaned.

### 2.3 Bioinformatic analysis

#### 2.3.1 Gene data acquisition

Candidate gene sets associated with the target toxicants were retrieved from the GeneCards database (https://www.genecards.org/). Genes with a relevance score >5 and classified as protein-coding genes were selected (Xing et al., 2026; Liu et al., 2026). Using the same parameters, an additional set of candidate genes related to cleft lip and palate (orofacial clefts) was obtained from GeneCards. Gene names from both sets were standardized and unified to generate an input dataset for subsequent intersection analysis.

A Venn diagram was generated using R software (version 4.3.1) and the Venn package (version 0.2.6) to visualize the overlap between the target toxicant- and orofacial cleft-related gene sets (Chen and Boutros, 2011; Team, 2023). The intersecting genes were defined as shared candidate genes associated with both the target toxicant and orofacial clefts and served as the fundamental input for subsequent protein–protein interaction (PPI) network analysis and enrichment analysis.

#### 2.3.2 PPI network construction

The intersection gene set was submitted to the STRING database to construct a PPI network (Szklarczyk et al., 2023). The analysis was performed using the *Homo sapiens* species background, and the interaction pairs were filtered using the default confidence threshold. The resulting PPI network was used to identify hub genes (confidence score = 0.4) (Wu et al., 2026). The PPI network generated by STRING was imported into Cytoscape, and topological analysis was performed using the cytoHubba plugin (Shannon et al., 2003; Chin et al., 2014). The degree algorithm was applied to calculate the degree centrality of each node, and all nodes were ranked in descending order of their degree values (Shannon et al., 2003; Chin et al., 2014). The top 20 hub genes were prioritized by degree of centrality as key candidate genes for subsequent pathway enrichment analysis.

#### 2.3.3 Pathway enrichment analysis

Kyoto Encyclopedia of Genes and Genomes (KEGG) pathway enrichment analysis of the top 20 hub genes was conducted using the enrichKEGG function in the clusterProfiler R package (Ogata et al., 1999; Yu et al., 2012). Significant pathways were identified using multiple-testing-corrected significance thresholds (the default method implemented in ClusterProfiler), and the top 10 most relevant pathways were reported (Benjamini and Hochberg, 1995).

## 3 Results

### 3.1 Number of publications

Figure 2 shows the continuous increase in the annual number of publications on NSCL/P genetic research. A total of 1,511 publications related to the present study were identified between 1990 and 2026 using comprehensive database retrieval and manual screening. The period from 1990 to 2000 witnessed a gradual increase in the number of annual publications, with the cumulative number exceeding 100 for the first time in 2000. Remarkable growth in publication output was observed after 2003. Subsequently, two explosive growth phases in the average annual number of publications were recorded between 2018 and 2021. This trend suggested a steadily increasing focus on NSCL/P genetics, which has developed into an active, rapidly expanding research field.

**FIGURE 2:**
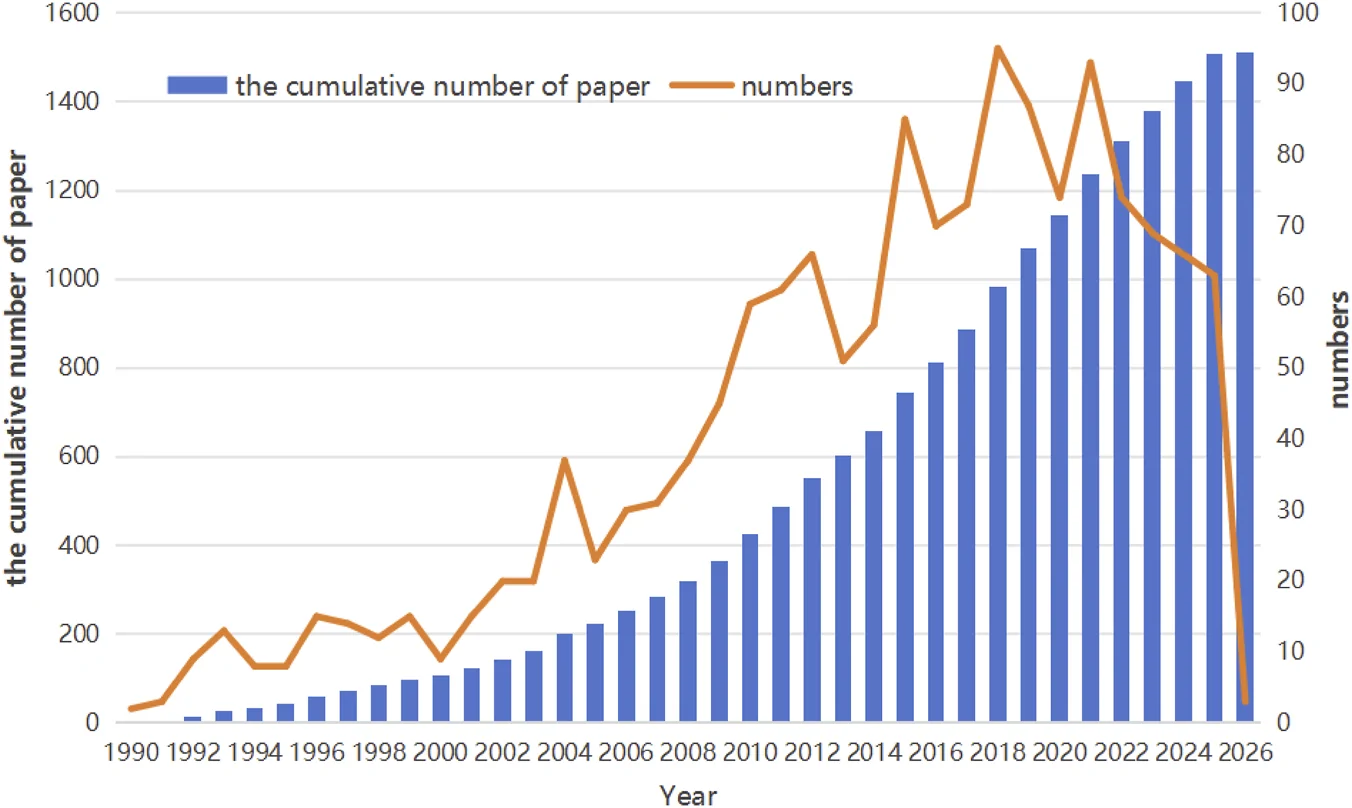
Annual and cumulative publication output trends in the genetics of NSCL/P, 1990–January 2026.

### 3.2 Contribution of countries

A total of 86 countries and regions contributed to the global publication output on NSCL/P genetics (Figure 3A). The top 10 most productive countries by publication volume are summarized in Table 1. The United States ranked first with 538 publications (35.6% of the total), followed by China (*N* = 365, 24.2%) and Brazil (*N* = 141, 9.3%). Regarding citation impact, the United States also ranked highest, with 24,752 citations, followed by the United Kingdom (*N* = 9,577) and China (*N* = 6,487). Notably, despite its lower publication count compared to the United States, China, and Brazil, the United Kingdom exhibited a high average number of citations per publication (*N* = 72.01), which was markedly higher than those of the United States (*N* = 46.01), China (*N* = 17.77), and Brazil (*N* = 30.36). These observations suggested that the research output from the United Kingdom had a relatively strong academic impact and has attracted extensive attention in this field.

**FIGURE 3:**
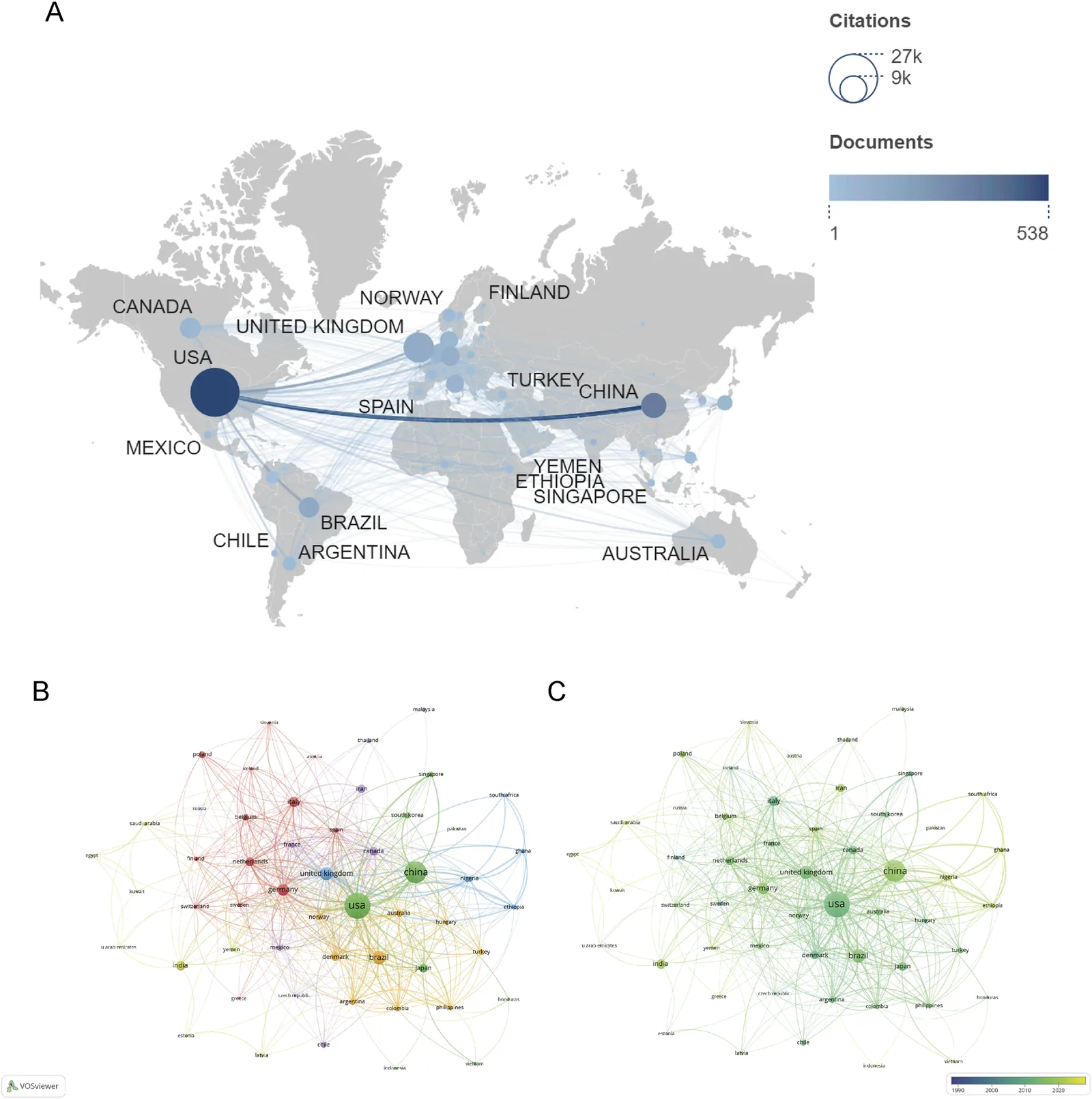
**(A)** Geographical distribution of global publications on genetics of NSCL/P. **(B)** Network visualization map of the countries with more than 10 publications. **(C)** Overlay visualization map.

**TABLE 1:** The 10 most productive countries in genetic research on NSCL/P.

Country	Count	Centrality	Citations
United States of America	538	0.58	24,752
China	365	0.02	6,487
Brazil	141	0.07	4,281
United Kingdom	133	0.16	9,577
Germany	108	0.1	3,754
Italy	83	0.05	3,318
India	72	0.05	450
Japan	62	0.11	2,390
Netherlands	59	0.08	3,361
Canada	58	0.15	4,528

A minimum publication threshold of 10 publications per country was applied to construct the co-authorship network, and 53 countries were identified using VOSviewer (Figure 3B). Intensified international research collaboration among these countries occurred primarily after 2005 (Figure 3C). Among the five most productive countries, China has recently shown a marked increase in collaborative research.

### 3.3 Contributions by institutions

In terms of institutional distribution, the top 10 most productive institutions in NSCL/P genetics were located in the United States, China, Italy, Germany, and Brazil (Figure 4A). A minimum threshold of 10 publications was applied, and 90 institutions with collaborative authorship patterns were identified using VOSviewer to form 10 clusters (Figure 4B). In addition to the top 10 core institutions, several other institutions have gained increasing influence recently. Notable examples include the University of Puerto Rico, the University of Lagos, Emory University, University Hospital Bonn, Addis Ababa University, and Xinjiang Medical University.

**FIGURE 4:**
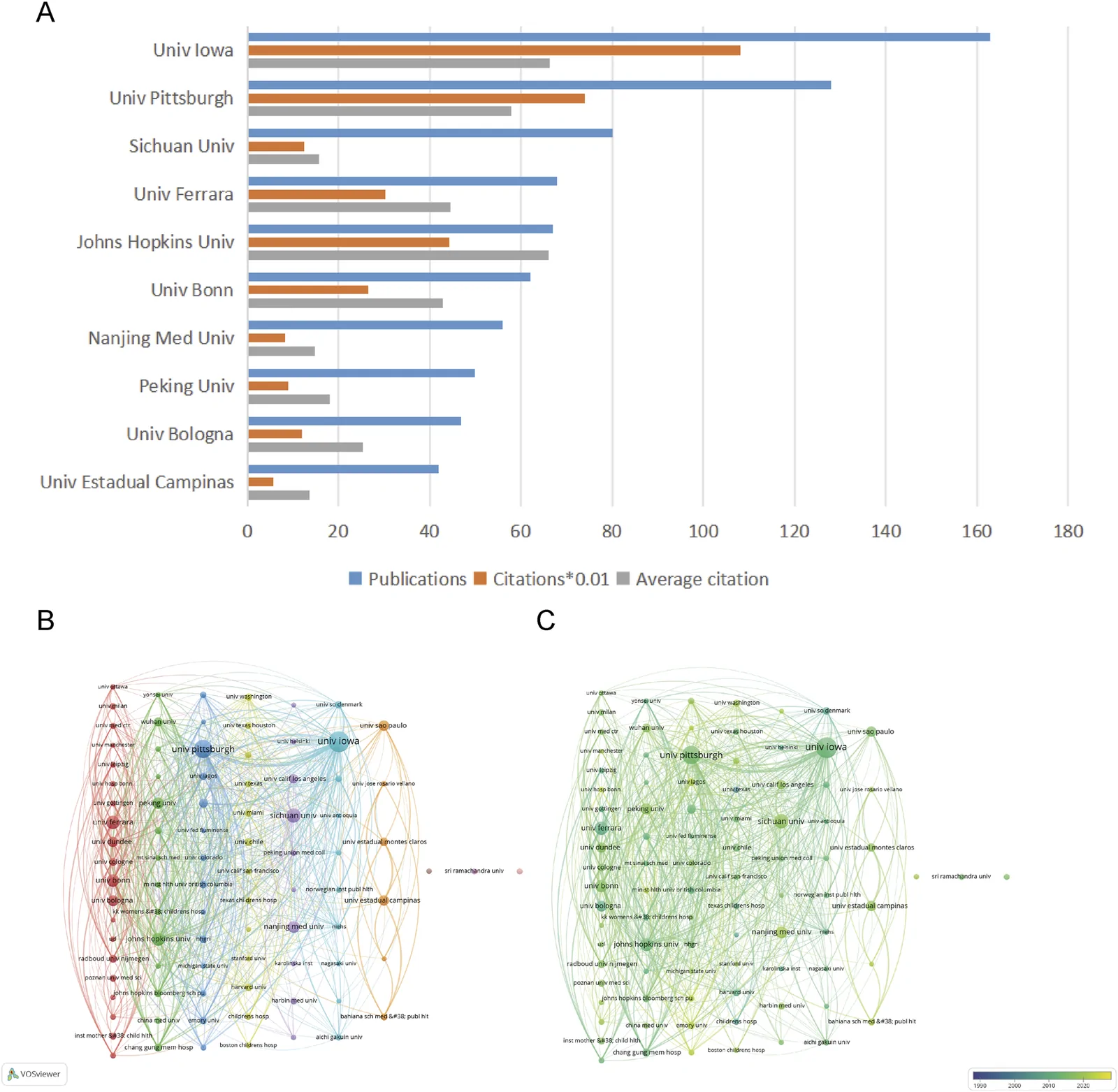
**(A)** The 10 most productive institutions in terms of total publications, total citation, and average citations. **(B)** Network visualization map of institutions. **(C)** Overlay visualization map of institution.

### 3.4 Contributions of prolific authors and co-cited authors

The author records retrieved for this bibliometric assessment were compiled into a final dataset containing 6,061 contributors. The author productivity distributions were evaluated according to Lotka’s law (Figure 5A). Most contributors (70.20%) had only one published output, 13.50% had two publications, and only 5.20% had three publications. The 10 most productive authors in the field of NSCL/P genetics are listed in Table 2. Most of the top 10 co-cited authors were from the United States, with the remainder originating from China, Germany, Italy, or Denmark. Murray, Jeffrey C., from the University of Iowa, ranked first with 93 publications (6.2% of the total), followed by Marazita, Mary L., from the University of Pittsburgh (*N* = 89, 5.9%), and Beaty, Terri H., from Johns Hopkins University (*N* = 66, 4.4%). These three authors also had the highest citation counts: Murray Jeffrey C. (*N* = 7,723), Marazita, Mary L. (*N* = 5,167), and Beaty, Terri H. (*N* = 3,673).

**FIGURE 5:**
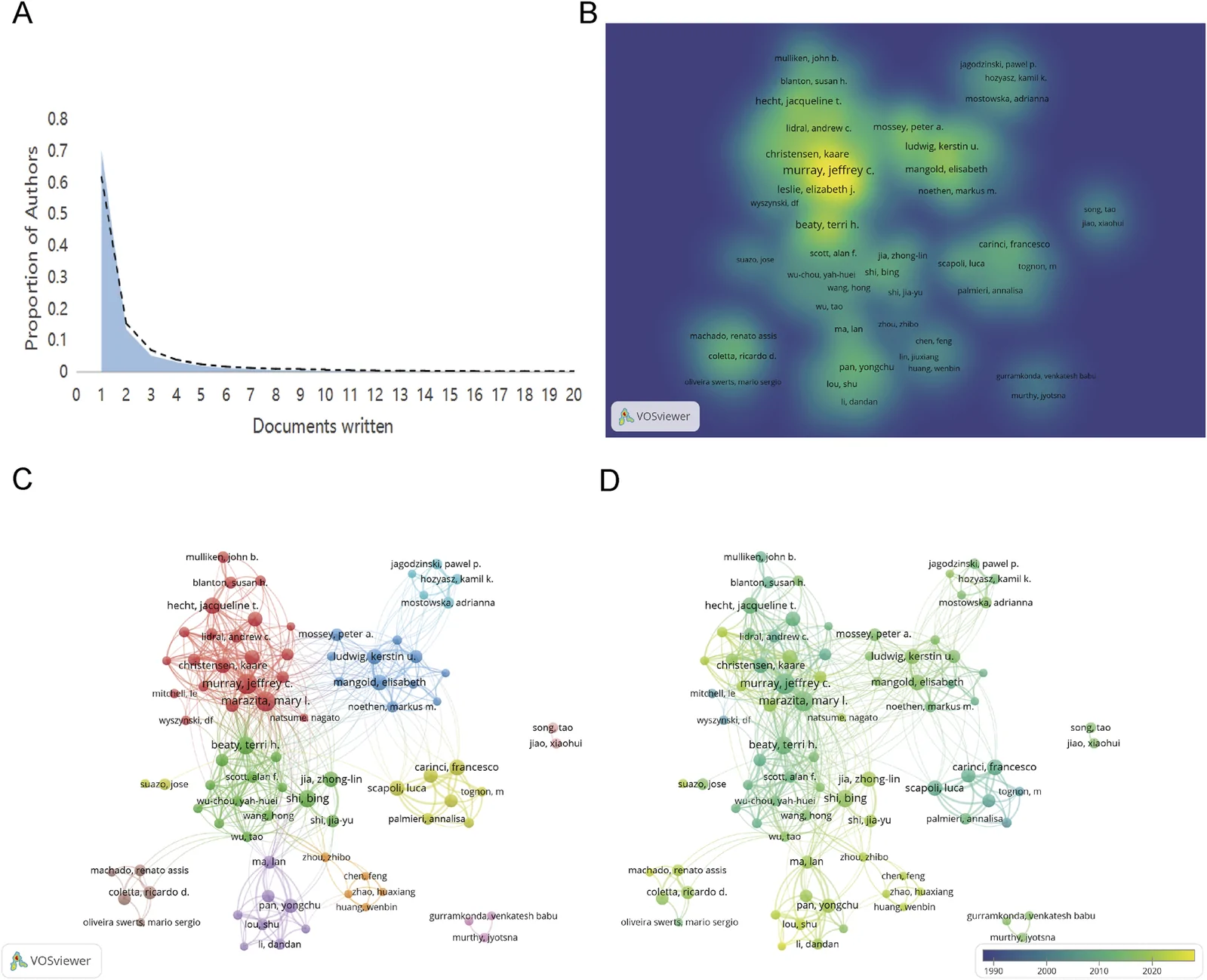
**(A)** Scientific productivity of authors based on Lotka’s law. **(B)** Density visualization map of authors. **(C)** Network visualization map of authors. **(D)** Overlay visualization map of authors.

**TABLE 2:** The 10 most productive authors in genetic research on NSCL/P.

Author	% of 1,511	Documents	Citations	Institution
Murray, Jeffrey c	6.15%	93	7,723	University of Iowa
Marazita, Mary l	5.89%	89	5,167	University of Pittsburgh
Beaty, Terri h	4.37%	66	3,673	Johns hopkins university
SHI, Bing	4.10%	62	604	Sichuan university
Hecht, Jacqueline t	3.51%	53	1,599	The university of Texas health science center at Houston
Mangold, Elisabeth	3.18%	48	1,170	University of bonn
Carinci, Francesco	3.11%	47	1,236	University of ferrara
Christensen, Kaare	2.85%	43	2035	University of southern Denmark
Jia, zhong-lin	2.85%	43	368	Sichuan university
Ludwig, Kerstin u	2.85%	43	1,089	Life & brain (Germany)
Scapoli, Luca	2.85%	43	1,141	University of ferrara

Figure 5B presents the co-authorship network constructed using VOSviewer, with a minimum threshold of 10 publications per author, yielding 84 eligible authors. The resulting visualization identified 10 distinct author clusters distinguished by color, revealing close collaborative connections among the researchers. Notably, pairwise co-authorship relationships were observed among the four most productive authors. Furthermore, the three authors with the highest citation counts were included in the same cluster, which displayed a higher average citation impact than the other groups (Figure 5C). Figure 5D presents a temporal analysis of the co-authorship relationship. Scholars such as Mitchell, L.E., and Wyszynski, D.F. emerged as key contributors during the early period (1990–2000); however, their collaborative networks remained relatively independent. During the intermediate period (2001–2015), Murray, Jeffrey C., Beaty, Terri H., and Scapoli, Luca served as central figures, and collaboration networks expanded rapidly, accompanied by a substantial increase in cross-institutional and -national cooperation. In the recent stage (2016–2026), numerous new researchers have emerged, represented by Leslie, Elizabeth J., Ma, Lan, and Pan, Yongchu, with collaborative networks expanding further into East Asia.

### 3.5 Research disciplines

The results of the journal dual-map overlay reveal the dynamics and trends in disciplinary development. The genetic research on NSCL/P spans multiple disciplines (Figure 6). The visualization was divided into two sections: the left zone represents citing journals, indicating an evolving research frontier, and the right zone represents cited journals, forming the knowledge base. Lines of different colors were used to distinguish the various disciplines. The citing journals mostly covered disciplines #2 (Medicine, Medical, and Clinical), #4 (Molecular, Biology, and Immunology), and #9 (Dentistry, Dermatology, and Surgery). In contrast, the cited journals were primarily distributed within disciplines #2 (Environmental, Toxicology, and Nutrition), #5 (Health, Nursing, and Medicine), #8 (Molecular, Biology, and Genetics), and #14 (Dermatology, Dentistry, and Surgery).

**FIGURE 6:**
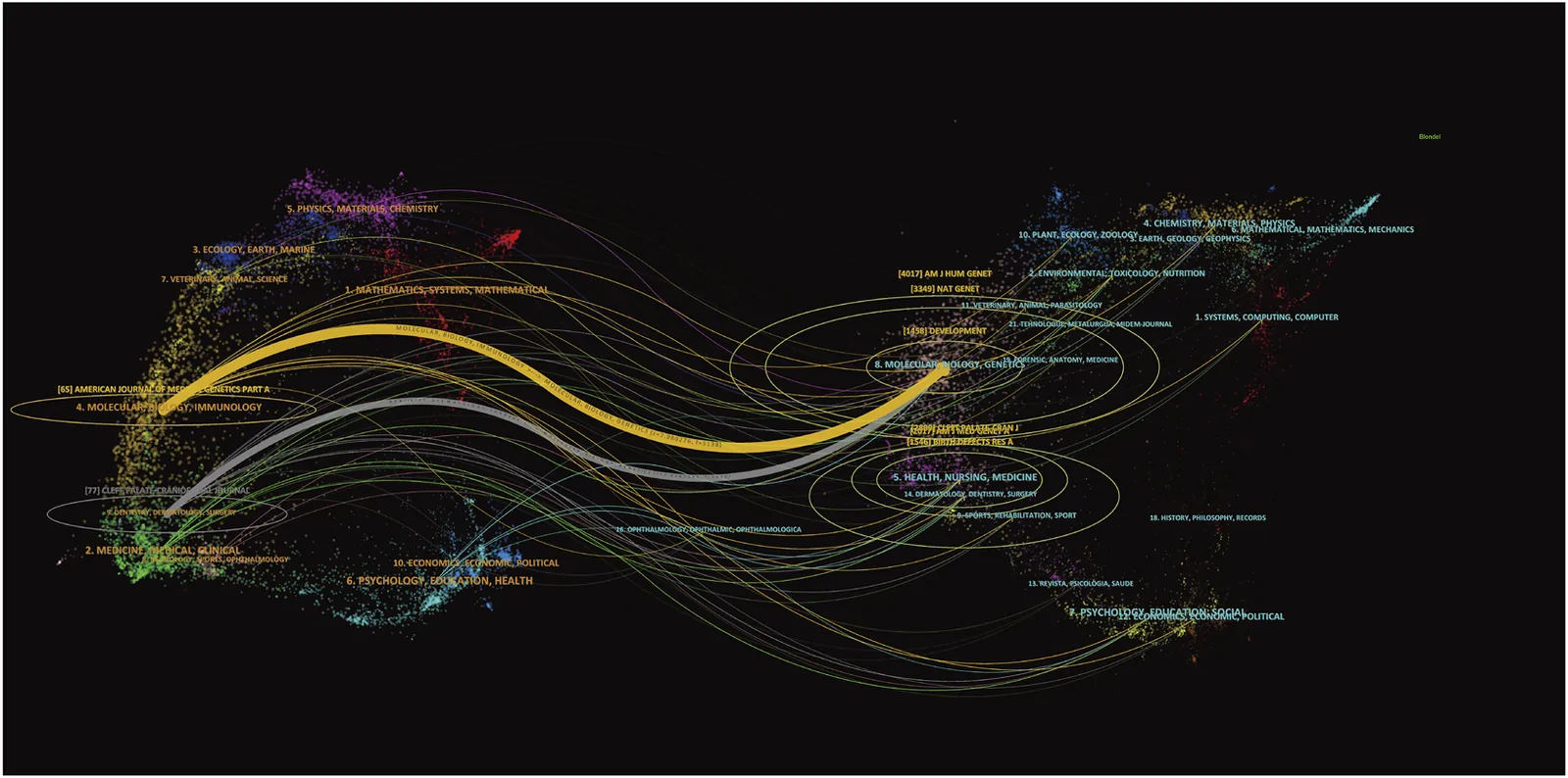
Dual-map overlay visualization of journals in the field of NSCL/P genetics.

### 3.6 Contribution of journals

A total of 383 journals have published articles or reviews in this field. The 10 most productive journals in this field are listed in Table 3, along with their total publication volumes and citation counts. The journal with the largest number of publications was Cleft Palate Craniofacial Journal (*N* = 154, 10.2% of the total), followed by American Journal of Medical Genetics Part A (*N* = 65, 4.3%) and Birth Defects Research Part A-Clinical and Molecular Teratology (*N* = 57, 3.8%). Simultaneously, the study period witnessed a notable surge in annual publication counts across numerous journals, particularly in 2010 (Figure 7A). Among the top 10 most productive journals, the Cleft Palate Craniofacial Journal has maintained a consistently high annual publication volume. Before 2000, this journal had already become a prominent publication, together with the American Journal of Human Genetics, both of which were recognized as long-standing core journals. From 2001 to 2015, the American Journal of Medical Genetics Part A and Birth Defects Research Part A: Clinical and Molecular Teratology exhibited a marked increase in publication output. Since 2016, journals such as Oral Diseases and Birth Defects Research have shown increased activity in their temporal heatmap visualizations, suggesting a rising publication output in these journals.

**TABLE 3:** The 10 most productive journals for genetic research on NSCL/P.

Journal	Documents	Citations	Jcr (2025)	If (2025)
Cleft palate craniofacial journal	154	3,022	Q3	1.7
American journal of medical genetics part a	65	2061	Q4	1.7
Birth defects research part a-clinical and molecular teratology	57	1,686	-	-
Oral diseases	44	801	Q1	3.1
Journal of dental research	36	1,096	Q1	6.6
Archives of oral biology	29	264	Q2	2.3
Birth defects research	27	367	Q3	2.0
International journal of pediatric otorhinolaryngology	26	359	Q2	1.6
American journal of human genetics	25	2,245	Q1	7.7
Human molecular genetics	25	2020	Q2	3.1
Journal of craniofacial surgery	25	355	Q3	1.1

**FIGURE 7:**
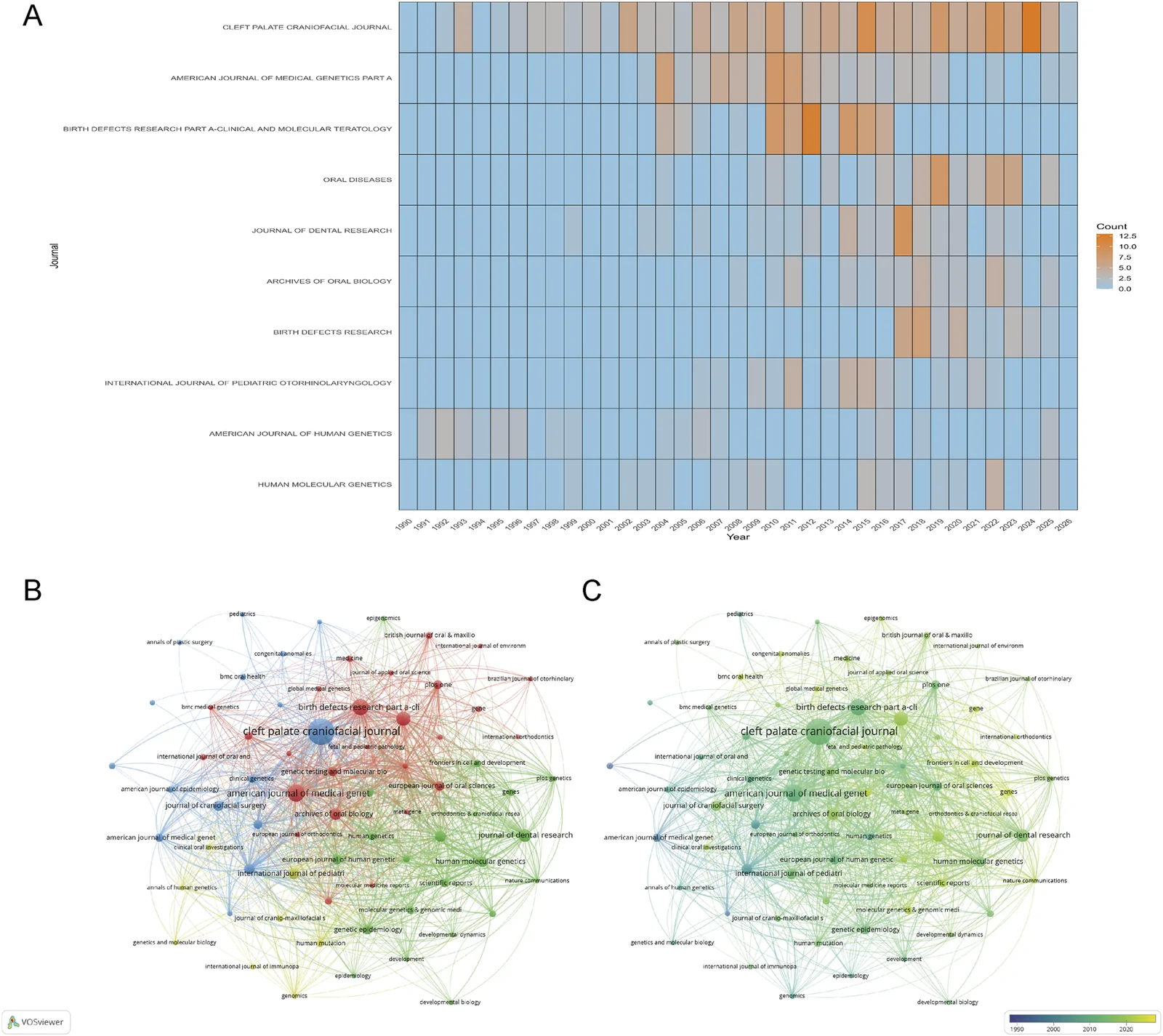
**(A)** Heat map of annual publications in the top 10 journals. **(B)** Network visualization map of journals. **(C)** Overlay visualization map of journals.

A minimum publication threshold of five publications was set, and 71 eligible journals were included in the network construction using VOSviewer. Figures 7B,C present the resulting network, which reflects the strength of inter-journal collaborations. A temporal evolution analysis identified four major journal clusters in the field of research. Among these clusters, the blue cluster represents the earliest-formed group, comprising influential journals in craniofacial surgery, including the Cleft Palate Craniofacial Journal and the Journal of Craniofacial Surgery. In contrast, the green cluster represents an emerging group of journals focused on genetic research, such as Nature Communications and the European Journal of Human Genetics. The four clusters were highly interwoven, demonstrating an evolutionary trend from fundamental clinical research to the expansion of basic science, and then to interdisciplinary integration. This pattern highlighted the multidisciplinary features and knowledge translation characteristics of NSCL/P genetic research.

### 3.7 References co-citation, clustering, timeline, and bursts

The 10 articles with the highest citation frequencies are listed in Table 4. The most frequently cited article, titled “Cleft lip and palate: Understanding genetic and environmental influences,” was published in *Nature Reviews Genetics* in 2011. A co-citation analysis of the literature was performed to clarify the intellectual landscape and track the progress of genetic research in NSCL/P. Figure 8A displays 23 major nodes with the most cited references published between 2004 and 2019. Figure 8B shows distinct clusters of co-cited references that classify the literature on NSCL/P genetics into several thematic categories. A total of 20 clusters were identified in this figure, with the first labeled “0# genome-wide association study,” the second “1# case-control study,” and the third “2# maternal smoking.” The themes of these clusters correspond to research hotspots at different developmental stages in the field of NSCL/P genetics.

**TABLE 4:** The top 10 co-citation references related to genetic research of NSCL/P.

Rank	Title	Doi	Year	First author	Journal	Citations
1	Cleft lip and palate: Understanding genetic and environmental influences	10.1038/nrg2933	2011	DIXON MJ	NAT REV GENET	129
2	A genome-wide association study of cleft lip with and without cleft palate identifies risk variants near MAFB and ABCA4	10.1038/ng.580	2010	BEATY TH	NAT GENET	108
3	Genome-wide association study identifies two susceptibility loci for nonsyndromic cleft lip with or without cleft palate	10.1038/ng.506	2010	MANGOLD E	NAT GENET	98
4	Genome-wide analyses of non-syndromic cleft lip with palate identify 14 novel loci and genetic heterogeneity	10.1038/ncomms14364	2017	YU YQ	NAT COMMUN	95
5	Genome-wide association study identifies a new susceptibility locus for cleft lip with or without a cleft palate	10.1038/ncomms7414	2015	SUN YM	NAT COMMUN	89
6	Genome-wide meta-analyses of nonsyndromic cleft lip with or without cleft palate identify six new risk loci	10.1038/ng.2360	2012	LUDWIG KU	NAT GENET	84
7	Key susceptibility locus for nonsyndromic cleft lip with or without cleft palate on chromosome 8q24	10.1038/ng.333	2009	BIRNBAUM S	NAT GENET	81
8	Genome-wide meta-analyses of nonsyndromic orofacial clefts identify novel associations between FOXE1 and all orofacial clefts, and TP63 and cleft lip with or without cleft palate	10.1007/s00439-016-1754-7	2017	LESLIE EJ	HUM GENET	75
9	A multi-ethnic genome-wide association study identifies novel loci for non-syndromic cleft lip with or without cleft palate on 2p24.2, 17q23 and 19q13	10.1093/hmg/ddw104	2016	LESLIE EJ	HUM MOL GENET	74
10	Cleft lip and palate	10.1016/s0140-6736(09)60695-4	2009	MOSSEY PA	LANCET	70

**FIGURE 8:**
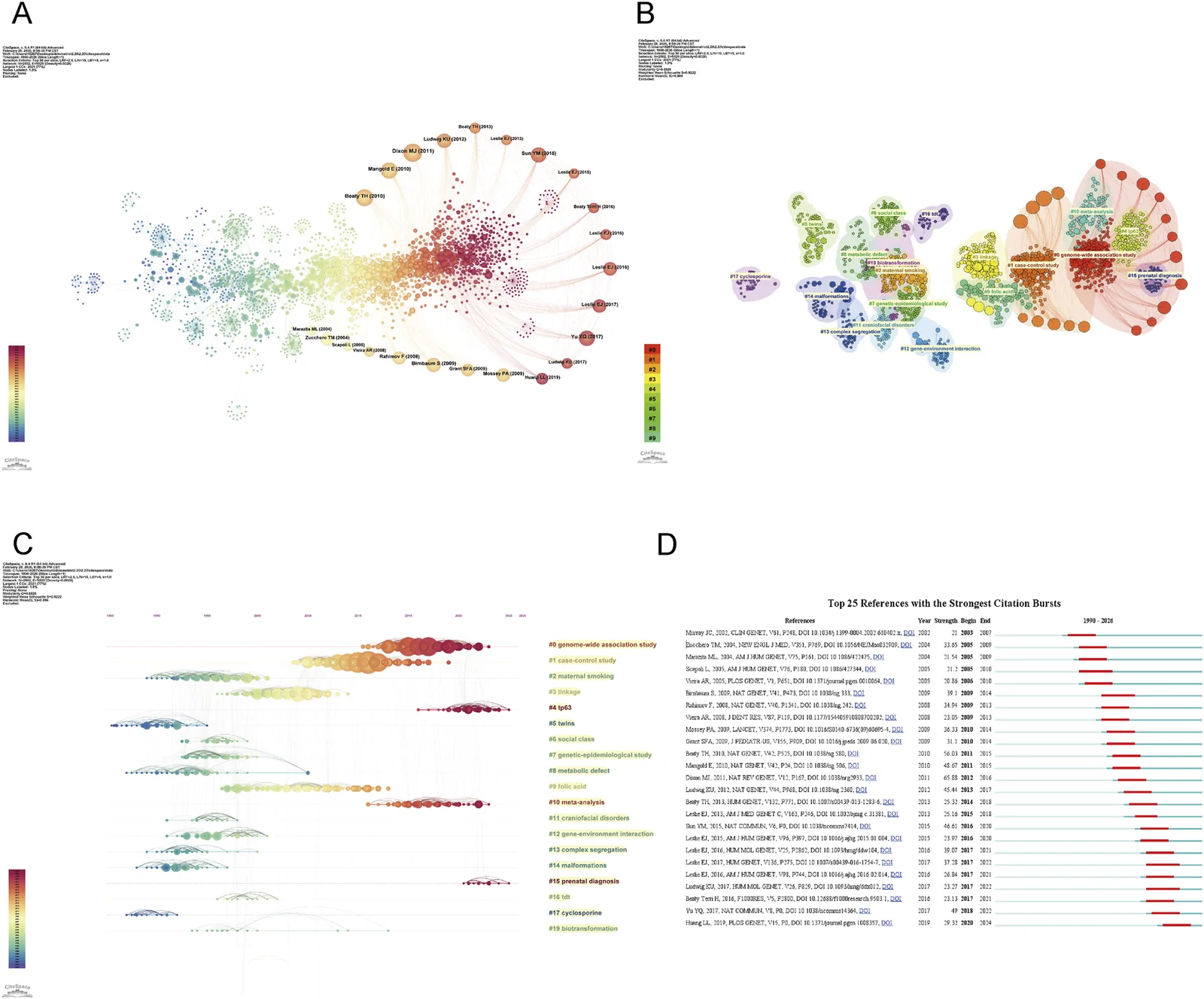
**(A)** Visualization of literature co-citations in genetics studies of NSCL/P. **(B)** Literature co-citations clustering in genetics studies of NSCL/P. **(C)** Timeline view of co-citations clusters, showing the chronological evolution of research themes. **(D)** The top 25 references with the strongest citation bursts.

A timeline of the co-citation reference clusters is shown in Figure 8C. Each cluster is represented by a horizontal line of nodes, with the research theme defined by the label on the right. The node size is proportional to the citation frequency. The chronological distribution, ranging from early fundamental topics on the left to emerging research fronts on the right, reflected the intellectual evolution of the research field. Notably, several prominent recent clusters, including “0# genome-wide association study,” “#4 tp63,” “#10 meta-analysis,” and “#15 prenatal diagnosis,” are identified, which represent the core research priorities of current NSCL/P genetics investigations. The results are shown in Figure 8D. The green line denotes the overall study period spanning 1990 to 2026, whereas the red segments mark the detected burst intervals. Notably, the article “Cleft lip and palate: Understanding genetic and environmental influences” (2012–2016, intensity 65.88), authored by Dixon in 2011, exhibited the highest burst intensity.

### 3.8 Keywords co-occurrence, clusters, and bursts

From the 1,511 retrieved publications, 4,488 keywords were screened and extracted for subsequent analysis. Table 5 lists high-frequency genetic keywords used in this field. The most frequently detected genetic keywords included *IRF6* (*N* = 167), *MTHFR* (*N* = 126), and *TGFA* (*N* = 115).

**TABLE 5:** The top 10 hotspot genes in genetic studies of NSCL/P.

Rank	Gene	Count
1	*IRF6*	167
2	*MTHFR*	126
3	*TGFA*	115
4	*MSX1*	74
5	*TGFB3*	38
6	*FOXE1*	23
7	*MAFB*	19
8	*VAX1*	16
9	*BMP4*	12
10	*CDH1*	11

A keyword co-occurrence analysis was conducted to explore the thematic framework of genetic research on NSCL/P, thereby contributing to a refined division of specific research topics in this field. The keyword co-occurrence network is illustrated in Figure 9A, and four independent clusters were identified via network analysis. Cluster 1 (blue cluster): genetic analysis methodologies, including association, linkage analysis, locus, and candidate genes. Cluster 2 (green cluster): developmental biology and phenotype, including phenotype, morphology, hypodontia, congenital malformations, defects, surgery, quality of life, parents, and relatives. Cluster 3 (yellow cluster): epidemiology and environmental factors, including maternal smoking, alcohol consumption, folic acid, metabolism, birth defects, prevention, polymorphism. Cluster 4 (red cluster): molecular mechanism of NSCL/P, including mutations, variants, genome-wide, molecular genetics.

**FIGURE 9:**
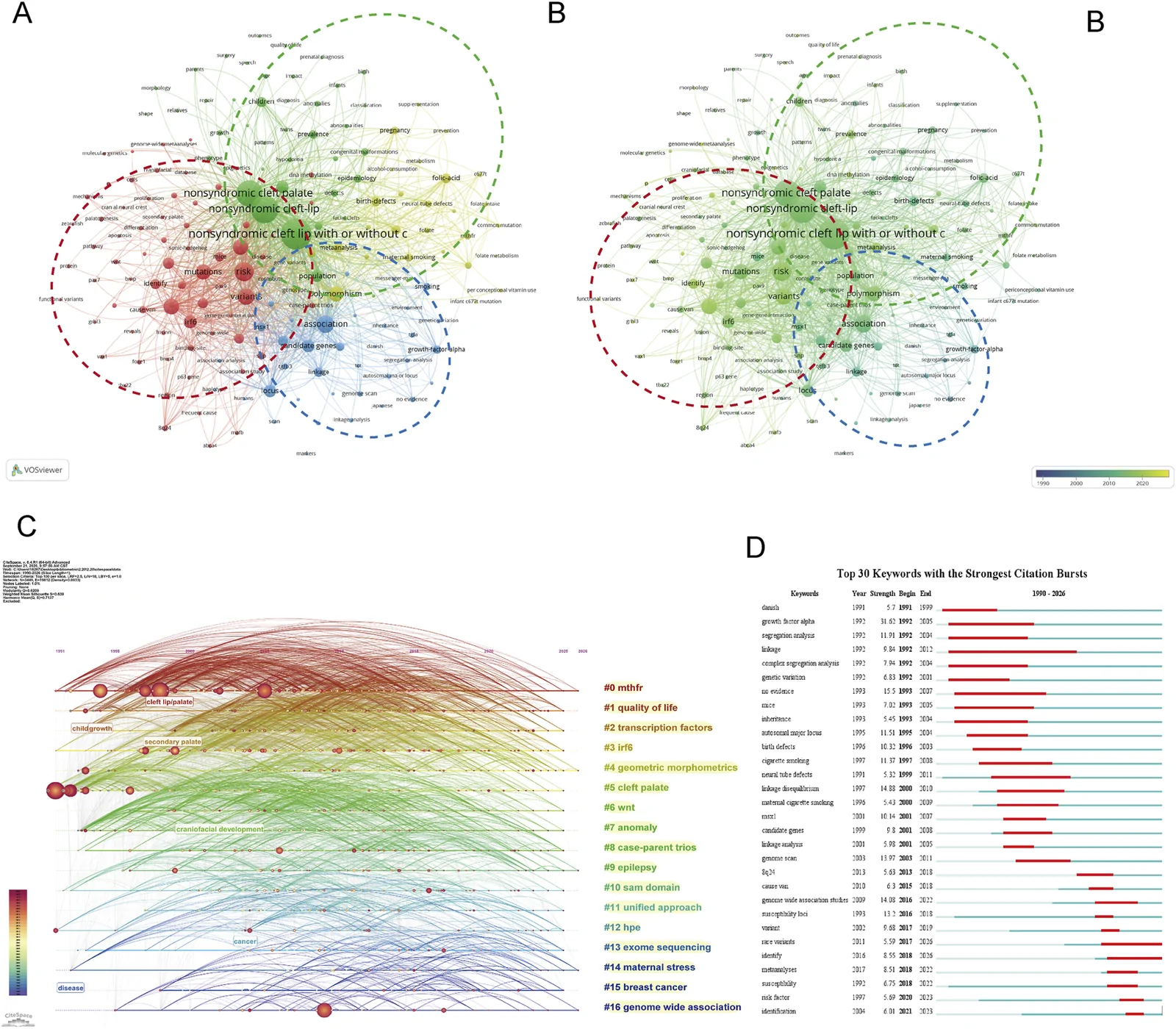
**(A)** Network map of keywords for genetics studies of NSCL/P. **(B)** Overlay visualization map of keywords. **(C)** Timeline view of keyword cluster. **(D)** The top 30 keywords with the strongest citation bursts.

The temporal evolutionary trends of research hotspots corresponding to the four keyword clusters are shown in Figure 9B. The blue cluster presented the most active research performance in the early stage (1990–2000). During the intermediate period (2001–2015), research interests shifted to themes included in the green and yellow clusters. In contrast, the red cluster played a dominant role in research activity in the recent stage (2016–2026). Longitudinal insight into the temporal trends and peak periods of the core research keywords was provided through timeline visualization. After removing disease terms directly associated with NSCL/P, several clusters with the longest research histories were identified (Figure 9C), including #0 (mthfr), #1 (quality of life), and #3 (irf6). All these keywords were regarded as active research topics since 1990. Additionally, new research fronts have been created using several emerging keywords, including exome sequencing, maternal stress, and genome wide association. A burst detection analysis was performed to identify emerging research hotspots at specific intervals. The 30 keywords with the strongest citation bursts are shown in Figure 9D.

### 3.9 Cross-database validation with Scopus

We conducted cross-validation using Scopus data to assess publication trends in NSCL/P genetic research and to mitigate potential biases inherent to a single database. In total, 1,457 papers were retrieved using an identical search strategy. Figure 10A compares the annual publication outputs of WoSCC and Scopus and shows parallel growth trajectories. Figures 10B,C show keywords that appeared no fewer than 25 times. The green cluster (1990–2015) focused on genetic methodology, population studies, and developmental genetic phenotypes, including associations, genetic markers, genetic linkages, *IRF6*, *MSX1*, genetic models, and inheritance. The blue cluster (1995–2015) centered on epidemiology and environmental risk factors, such as environmental exposure, folic acid, alcohol consumption, and smoking. The red cluster (2010–2026) focused on the molecular mechanisms of NSCL/P, including gene mutations, embryonic development, animal models, gene sequencing, gene upregulation, clinical investigations, and bioinformatics. The three keyword clusters identified in Scopus confirmed the findings in WoSCC, reflecting the developmental trajectory of NSCL/P genetic research.

**FIGURE 10:**
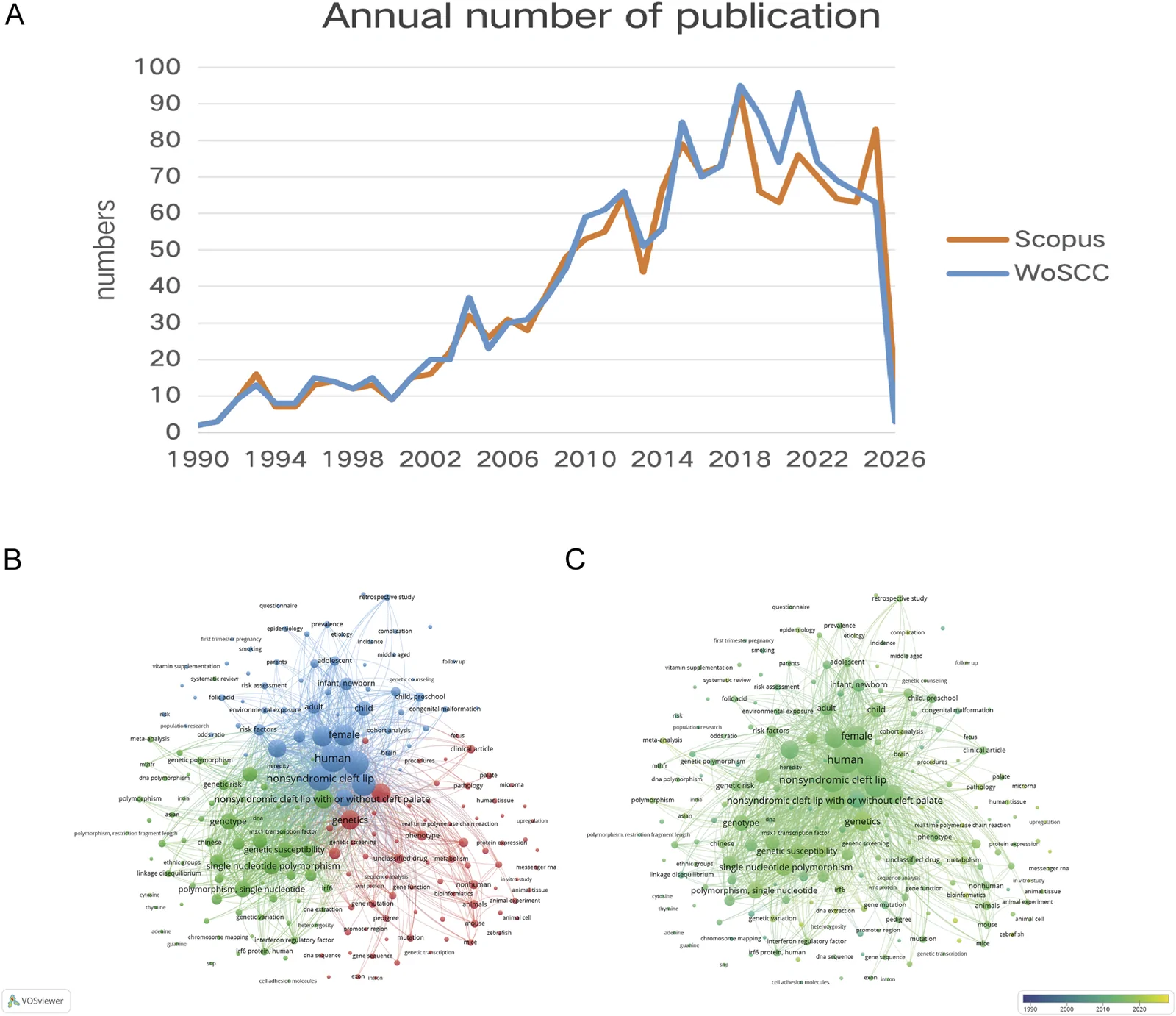
**(A)** Annual publication trends in WoSCC and Scopus. **(B)** Network map of keywords for genetics studies of NSCL/P (Scopus). **(C)** Overlay visualization map of keywords (Scopus).

### 3.10 Alcohol- and nicotine-induced targets in NSCL/P and associated biological effects

To systematically explore the development of research on environmental toxicant-induced NSCL/P, 823 alcohol-and 252 nicotine-associated target genes were retrieved from the GeneCards database. In parallel, 2,745 NSCL/P-associated target genes were collected from the GeneCards database. Intersectional analysis of these datasets identified 331 overlapping genes potentially involved in the pathogenesis of alcohol-induced NSCL/P and 85 overlapping genes implicated in nicotine-induced NSCL/P (Figures 11A,E).

**FIGURE 11:**
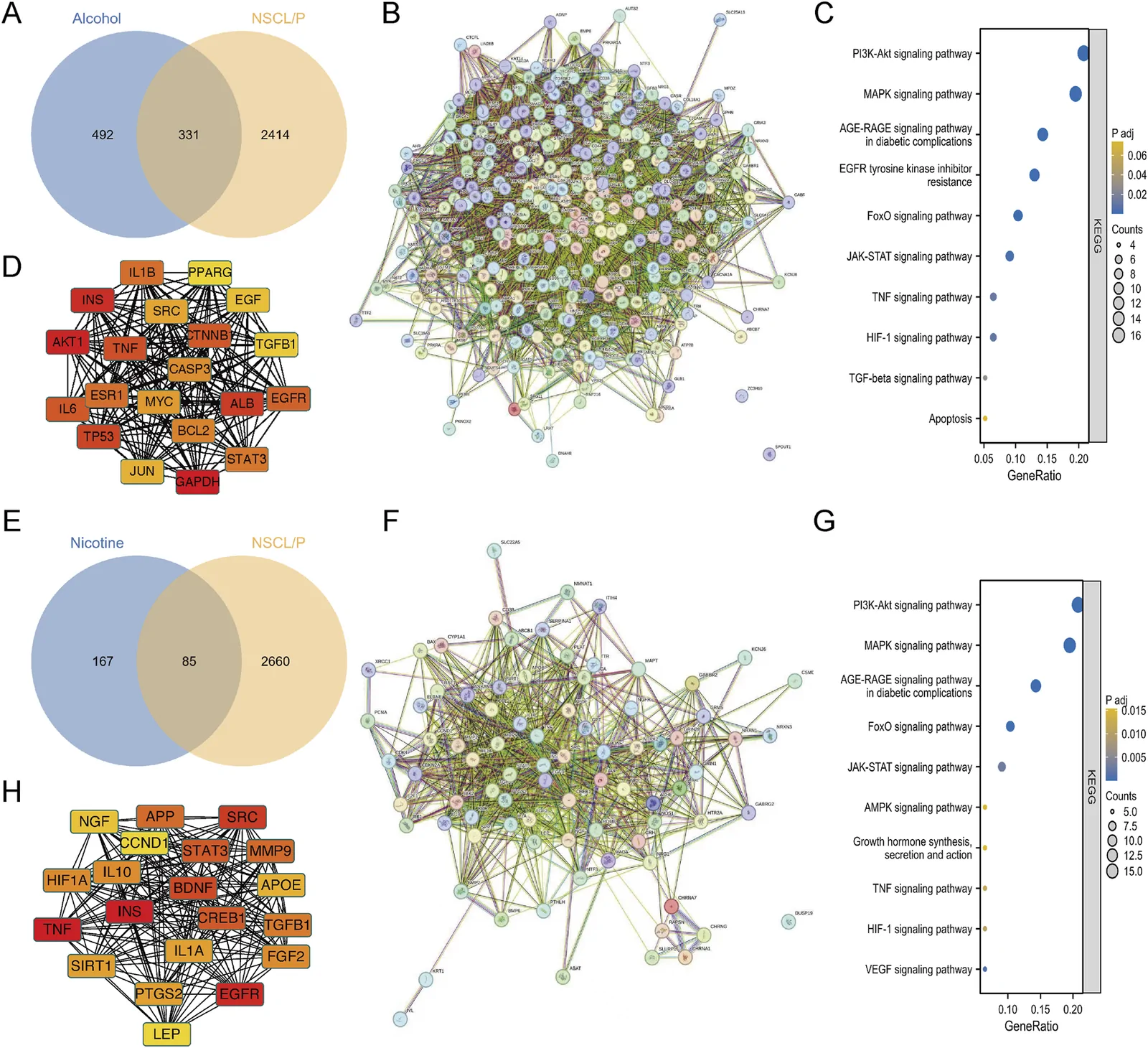
**(A)** Venn diagram analysis identifying potential target genes associated with both alcohol exposure and NSCL/P. **(B)** String database presentation of correlation between Alcohol-induced NSCL/P genes. **(C)** Results of KEGG pathway enrichment among Alcohol-induced NSCL/P. **(D)** Cytotrace analysis of the top 20 key gene nodes in Alcohol-induced NSCL/P genes. **(E)**Venn diagram analysis identifying potential target genes associated with both Nicotine exposure and NSCL/P. **(F)** String database presentation of correlation between Nicotine-induced NSCL/P genes. **(G)** Results of KEGG pathway enrichment among Nicotine-induced NSCL/P. **(H)** Cytotrace analysis of the top 20 key gene nodes in Nicotine-induced NSCL/P genes.

A PPI network was constructed for the two groups of NSCL/P-associated genes using the STRING database (Figures 11B,F). PPI network analysis revealed a synergistic interaction mode among overlapping target genes, with 331 alcohol-related overlapping genes forming a complex network centered on *AKT1*, *IL6*, and *GAPDH*. In contrast, the 85 nicotine-related overlapping genes formed a functional interaction network centered on *EGFR*, *TNF*, and *HIF1A* (Figures 11D,H).

Pathway enrichment analysis was performed using the KEGG database on overlapping gene sets between alcohol- or nicotine-associated targets and NSCL/P-related genes. Statistical enrichment was observed for both sets in the PI3K-Akt, MAPK, AGE-RAGE, FoxO, JAK-STAT, TNF, and HIF-1α signaling pathways (Figures 11C,G). Distinctly, alcohol-specific targets were enriched in the TGFβ signaling pathway and apoptosis, whereas nicotine-specific targets were enriched in the VEGF, AMPK, and growth hormone synthesis pathways (Figures 11C,G).

## 4 Discussion

### 4.1 Global research status and trends

The publication output of NSCL/P genetic research has maintained an increasing trend over the years, with a notable increase observed after 2003. Recently, while the annual output has remained high, the growth rate has stabilized after 2022. Taken together, these three observations—the acceleration after 2003, the explosive growth phase between 2018 and 2021, and the stabilized growth rate after 2022—suggest that the development of NSCL/P genetic research has proceeded through three successive methodological paradigms rather than a simple growth curve. The first acceleration coincided with the landmark identification of IRF6 as a landmark early major susceptibility gene through linkage and association strategies (Zucchero et al., 2004). This discovery transformed NSCL/P genetics from a field constrained by scarce *a priori* hypotheses into a gene-mapping discipline. The steepest growth between 2018 and 2021 followed the maturation of multi-ancestry GWAS meta-analyses and the widespread application of next-generation sequencing (Yu et al., 2017; Leslie et al., 2017). These advances expanded the documented catalog of susceptibility loci to over 40 and substantially broadened the accessibility of genomic screening. Notably, the reference with the strongest citation burst in the field—Dixon et al., 's 2011 synthesis—was a review rather than a discovery paper (Dixon et al., 2011). This pattern is characteristic of maturing research fields. In such fields, synthetic reviews become focal points of citation because empirical findings accumulate faster than they can be integrated. This shift marks the transition from discovery-driven to framework-driven research. Accordingly, the stabilized growth rate after 2022 should not be interpreted as waning interest. It indicates that common-variant discovery is approaching saturation and that the field is entering a consolidation phase. This interpretation is consistent with the progressive shift of citing journals toward clinical application and molecular-mechanism outlets.

In terms of the research discipline, a significant discrepancy between citing and cited journals was revealed. Citing journals are concentrated on clinical applications and molecular mechanism research, whereas cited journals cover basic disciplines, including genetics and environmental science. This pattern indicates that solid foundations in basic disciplines have been laid for NSCL/P genetic research, and a trend has emerged that has shifted from theoretical exploration to clinical translational practice. The co-citation analysis further supports this disciplinary distribution and illustrates the developmental trajectory of this field. In the early stages, studies focused on classical genetic epidemiological approaches, including twin studies and linkage analyses. In the middle period, priorities shifted toward case-control designs and gene–environment interaction analyses. Recently, GWAS has emerged as a mainstream approach, advancing candidate gene identification and the exploration of molecular mechanisms (Robinson et al., 2023). This evolving progress has enriched the theoretical framework underlying the genetic mechanisms of NSCL/P and laid the foundation for the establishment of preventive and therapeutic protocols.

Among countries and institutions, the United States ranked first in both publications and total citations. However, despite the larger publication volumes in these nations, lower average citations per publication were observed in the United States, China, and Brazil than in the United Kingdom. This discrepancy between productivity and impact has also been reported in previous bibliometric assessments of oral cleft research, suggesting that strengthened cooperation with leading international teams and improved research quality should be prioritized to advance the field (Xu et al., 2026). Scholars have established their research status as academic contributors, including Murray, Jeffrey C., Marazita, Mary L., and Beaty, Terri H., whose research priorities have centered on susceptibility gene mapping, gene–environment interaction studies, and clinical translation. Overlapping publication outputs across careers can be attributed to long-term collaborative partnerships. Co-authorship network visualization reveals that most collaborative activities remain confined to domestic teams. However, several institutions, including the University of Iowa, the University of Pittsburgh, and Sichuan University, have achieved high publication productivity and serve as key network nodes. This distribution pattern can be explained by the concentration of senior researchers at these institutions; further cross-regional cooperation is required to strengthen global academic communication.

### 4.2 Research keyword analysis

Cluster analysis of 178 keywords yielded four distinct clusters, which were mapped onto four interrelated thematic domains: genetic analytical approaches; developmental biology and phenotypic characteristics; epidemiology and environmental risk factors; and molecular mechanisms underlying the disease. Combined with the timeline graph of keywords and the top 30 burst terms, mthfr, and quality of life maintained a sustained high frequency from 1990 to 2026, thereby serving as persistent research hotspots across the field. Recently, the importance of keywords such as genome-wide association studies and exome sequencing have been increasingly prominent. The emergence of clusters related to comorbid conditions, such as epilepsy, and breast cancer, further indicates that NSCL/P genetics has recently expanded toward pleiotropic and mechanistic questions, which is consistent with the comorbidity analyses discussed below. These studies are closely linked to the exploration of molecular mechanisms in NSCL/P and are highly consistent with the clustering results derived in the current study. Through a systematic analysis of the four core clusters using a timeline visualization, this study identified hotspots and research trends in the genetics of NSCL/P.

Furthermore, the burst-detection analysis offers a fine-grained record of these paradigm shifts. Each burst term can be regarded as the signature of a methodological generation. The earliest bursts—segregation analysis, linkage, and complex segregation analysis—all began in 1992. They correspond to the era in which family-based studies formally established the complex polygenic architecture of NSCL/P. The second wave, represented by growth factor alpha (1992–2005), MSX1 (2001–2007), and candidate genes (2001–2008), marks the candidate-gene era. Notably, growth factor alpha exhibited the strongest burst strength (31.67). During this period, prior knowledge of craniofacial morphogens guided association testing. The third wave is clearly technology-driven. It comprises genome scan (2003–2011), 8q24 (2013–2018), and genome-wide association studies (2016–2022). Of note, the most recent bursts that remain active through 2026 are rare variants, identify, and identification. This pattern indicates that the research focus of the field has shifted from common-variant association toward the discovery and characterization of rare, potentially high-penetrance variants through exome and genome sequencing. This trajectory, extending from linkage analysis through candidate-gene studies and common-variant GWAS to rare-variant discovery, closely mirrors the methodological evolution of complex-disease genetics as a whole. NSCL/P genetics may therefore be regarded as a microcosm of the post-genomic era. In addition, the dominance of IRF6, MTHFR, and TGFA in keyword frequency is itself informative. IRF6 has remained at the forefront. It represents the largest-effect locus identified to date and a hub of subsequent functional studies. TGFA and MTHFR, by contrast, are legacy candidate genes whose early associations were only partially replicated in GWAS. Their sustained high frequency may therefore reflect citation inertia as much as current etiological importance. Keyword counts measure scholarly attention; they do not necessarily indicate biological centrality.

#### 4.2.1 Early stage (1990–2000): blue cluster (genetic analysis methodologies)

The blue cluster represents the most active research front during the early period, which focused on genetic analysis methodologies. Family-based segregation analysis has revealed that NSCL/P follows a complex polygenic inheritance pattern, with environmental factors modifying its effects (Farrall and Holder, 1992; Mitchell and Risch, 1992; Hecht et al., 1991). The single-gene inheritance hypothesis was excluded from this finding, and a solid theoretical foundation was provided for subsequent research. Linkage analysis has served as the primary tool for localizing susceptibility loci prior to the widespread application of GWAS (Cichon et al., 2009). Using this method, several chromosomal regions, including 19q13 and 2p13, were preliminarily identified as associated with NSCL/P across multiple populations (Wong et al., 2000; Field et al., 1994). Guided by the craniofacial developmental hypothesis, candidate genes, including *TGFA*, *MSX1*, and *BMP4*, have been preferentially screened (Ardinger et al., 1989; Satokata and Maas, 1994; Bennett et al., 1995). Association analysis revealed that polymorphic loci in *TGFA* and *MSX1* co-segregated with NSCL/P phenotypes in the familial pedigree (Feng et al., 1994). Although population-level heterogeneity was detected in some studies, the research paradigm of candidate gene screening followed by population validation was firmly established during this period (Schutte and Murray, 1999). This framework advances the characterization of known genes, facilitates the discovery of additional pathogenic genes, such as *IRF6*, and supports mechanistic investigations into the associated signaling pathways (Zucchero et al., 2004). Taken together, the lasting contribution of this early era was not any single susceptibility locus, but the establishment of NSCL/P as a tractable, genetically heterogeneous complex trait, without which the subsequent GWAS era could not have emerged.

#### 4.2.2 Middle stage (2001–2015): green cluster (developmental biology and phenotypes) and yellow cluster (epidemiology and environmental factors)

At mid-term, the research hotspots shifted to the green cluster, themed on developmental biology and phenotype, and the yellow cluster focused on epidemiology and environmental factors. Notably, the limitation of focusing solely on morphological descriptions of craniofacial development was overcome at this stage. Candidate gene screening and GWAS have been used to conduct large-scale explorations of phenotype–genotype correlations and to dissect the genetic heterogeneity of distinct phenotypic subtypes. This shift has driven a pivotal paradigm change in NSCL/P from a morphological description to the dissection of etiological mechanisms. Additionally, population-based association analysis, animal models, and cellular functional experiments were used to explore susceptible variants and the molecular regulatory mechanisms of classic candidate genes, including *IRF6*, *TGFB3*, and *MSX1* (Zhang et al., 2002; Park et al., 2007; Suazo et al., 2010; Rahimov et al., 2008). These genes do not act independently; they regulate craniofacial development through cross-pathway crosstalk and gene–gene synergistic interactions. This period was regarded as a pivotal stage in the development of NSCL/P genetics (Beaty et al., 2010). Research on the susceptibility landscape and pathogenic mechanisms of NSCL/P has significantly expanded through investigations of gene–environment interactions, phenotype–genotype associations, and GWAS. Genetic research on NSCL/P has shifted from single-dimensional genetic mapping to multidimensional integrated analysis, laying a solid foundation for subsequent molecular mechanistic studies and clinical translation.

#### 4.2.3 Recent stage (2016–2026): red cluster (molecular regulatory mechanism of NSCL/P)

Since 2015, the red cluster has remained at the forefront of NSCL/P genetic research. This phase is characterized by GWAS, mutations, and variants as core keywords. The functional and molecular mechanisms of key pathogenic genes, including *TP63* and *CDH1,* have been intensively investigated, and research outcomes have increasingly been translated into clinical applications, such as prenatal diagnosis and precision prevention. These findings demonstrate that research priorities in NSCL/P genetics have shifted toward the exploration of molecular mechanisms and clinical translational research. Candidate gene identification continues to expand the spectrum of susceptible loci beyond 40, with attention shifting toward regulatory mechanisms in non-coding regions (Jia et al., 2024; Ludwig et al., 2017; Sun et al., 2015; Avasthi et al., 2021; Ma et al., 2020). Single-cell and transcriptomic sequencing have been used to explore craniofacial development and the underlying molecular regulatory networks. A two-locus inheritance model based on epistatic antagonism was proposed, which differs from the conventional digenic inheritance model and further optimizes the polygenic synergistic pathogenic architecture of NSCL/P (He et al., 2025). Research has also been extended to comorbidity analyses, with associations investigated between NSCL/P and disorders, including hypothyroidism and cancer, providing a novel perspective for elucidating pathogenic mechanisms (Lidral et al., 2015; Raterman et al., 2025). Although clinical translation pathways and intervention strategies remain unclear, advances in transcriptomics are accelerating progress toward precision prevention (Yang et al., 2026). Noninvasive prenatal testing has been widely used for prenatal diagnosis of multiple congenital malformations (Dupras et al., 2022; Löwy, 2022). After further verification and optimization, this technology, combined with high-throughput next-generation sequencing, is expected to detect and analyze circulating cell-free DNA in maternal peripheral blood and noninvasively identify fetal NSCL/P-related genetic variants (Stanley et al., 2025; Schwammenthal et al., 2024). Precision prevention strategies that integrate genetic susceptibility and environmental risk factors are gradually improving. The persistence of identification-related keyword bursts into 2026 suggests that this mechanism-oriented phase remains in its discovery stage rather than having entered a mature synthesis stage, implying that the next inflection point of the field will depend on which of these molecular findings can be functionally validated and effectively translated into clinical practice.

### 4.3 Cross-database validation

Cross-validation with Scopus significantly strengthened the reliability of the results obtained from the WoSCC. Although the two databases differ in their publication counts, their annual publication trends show largely parallel curves and nearly identical growth patterns. Keyword comparisons further validated the reliability of the thematic patterns identified via the WoSCC and provided complementary insights. Based on the keyword co-occurrence analysis, we confirmed the evolutionary trajectory of NSCL/P genetic research since the launch of the Human Genome Project. The early stages primarily focused on genetic analysis methodologies and susceptibility locus mapping. Middle-stage research has shifted to environmental risk factors and craniofacial development. Recent studies have employed bioinformatics, gene sequencing, and other techniques to elucidate the underlying molecular mechanisms (Fang et al., 2025; Xiong et al., 2026). We further clarified the potential mechanisms of environmental risk factors and the progression of craniofacial development. The parallel evolution of keyword clusters in the WoSCC and Scopus not only confirms the robustness of our findings but also carries a substantive implication. The paradigm shifts identified in this study reflect the behavior of the scientific community rather than artifacts of any single indexing database. The three Scopus clusters—genetic methodology (1990–2015), epidemiology and environmental risk (1995–2015), and molecular mechanism (2010–2026)—recapitulate the same left-to-right transition observed in the WoSCC. Notably, the molecular-mechanism cluster overlaps both earlier clusters in time. This temporal overlap suggests that mechanism-oriented research is currently assimilating the earlier methodological traditions rather than replacing them. This cross-database concordance demonstrates that our bibliometric mapping reflects the authentic scholarly evolution in this field, rather than biases specific to individual databases.

### 4.4 Environmental risk factors were identified by combining bibliometrics and bioinformatics

The bibliometric findings delineate an evolutionary trajectory in NSCL/P genetic research from linkage analysis to multi-omics integration. Research on environmental risk factors has followed a course comparable to that of molecular network interrogation. However, the molecular bridge connecting exposure to malformations remains poorly characterized (Garland et al., 2020). GeneCards and STRING resources were integrated for the network toxicology analysis of alcohol- and nicotine-induced targets. Alcohol and nicotine were selected as exemplar exposures for three reasons supported by our bibliometric data. First, both exposures have consistently ranked among the most frequent environmental keywords across all 3 decades. Second, cigarette-smoking-related keywords produced the strongest environmental bursts in the dataset, signaling sustained etiological attention. Third, although alcohol and nicotine are the most extensively documented modifiable environmental risk factors for NSCL/P, the molecular mechanisms through which they perturb craniofacial development remain incompletely characterized. Closing this gap between epidemiological association and mechanistic understanding is precisely the objective of the present network analysis.

Enrichment analysis revealed seven shared cascades: PI3K-Akt, MAPK, AGE-RAGE, FoxO, JAK-STAT, TNF, and HIF-1α. While the PI3K-Akt and MAPK pathways direct craniofacial development, the remaining pathways have been implicated in convergent processes, including inflammation, oxidative stress, hypoxia, and apoptosis (Dinsmore and Soriano, 2018; Cai et al., 2022; Huang et al., 2011). STRING analysis identified *INS*, *TNF*, *TGFB1*, *STAT3*, and *EGFR* as shared hub nodes. Functionally, both teratogens may compromise epithelial proliferation and differentiation within the palatal shelves, preventing adequate outgrowth and medial edge epithelial adhesion during the critical developmental window. This convergence explains how distinct environmental conditions induce the funnel to form a unified NSCL/P phenotype.

Despite their shared pathogenic foundations, alcohol and nicotine exhibit markedly divergent signatures in specific regulatory contexts. Alcohol-related intersection targets showed selective enrichment in TGF-β signaling and apoptosis. TGF-β governs medial edge epithelial differentiation and basement membrane dissolution, whereas apoptosis provides programmed cell death required for palate fusion; both are indispensable for closure. This selective pattern suggests that alcohol preferentially disrupts the fusion program. Additionally, *IL6* and *TNF* are central regulatory molecules within the alcohol-specific network, supporting the inference that alcohol compromises embryonic microenvironmental stability by activating endogenous oxidative stress and persistent inflammatory stimulation (Dobrucki et al., 2024; Zou et al., 2026; Lussier et al., 2021).

In contrast, the nicotine-related intersection targets were selectively enriched for VEGF, AMPK, and growth hormone synthesis. Although HIF-1α and FoxO represent shared pathways between the two exposures, *HIF1A* is a critical functional gene in the nicotine-specific PPI network, and VEGF, an established downstream effector of HIF-1 signaling, was selectively enriched, implicating aberrant hypoxia-induced angiogenesis as a prominent pathogenic modality (Zhang et al., 2007; Shi et al., 2012; Küchler et al., 2018). These findings corroborate the view that maternal smoking contributes to localized hypoxic damage in the fetal craniofacial tissues (Juliá-Burchés et al., 2024; Lacerda et al., 2021). Nicotine-driven HIF-1α activation and FoxO perturbation may interfere with the spatiotemporal progression of facial process fusion. Therefore, the dual commonality and specificity highlight the need to advance from environmental association to the precise mechanistic elucidation of the gene–environment interplay in NSCL/P.

### 4.5 Limitations

This study had several limitations. First, the bibliometric data were primarily drawn from the WoSCC, with Scopus used only for cross-validation. Publications indexed exclusively in PubMed or EMBASE may have been overlooked. Restrictions on English-language articles may also have introduced language bias by excluding relevant studies published in other languages. Second, citation-based metrics naturally suffer from temporal lags, and ongoing indexing delays, particularly for 2026 publications, may have led to an underestimation of their current citation impact. Third, all bioinformatic findings were generated from publicly available databases. Therefore, the identified candidate genes and enriched pathways should be treated as computationally prioritized hypotheses that require experimental validation rather than as confirmed biological mechanisms.

In conclusion, this study summarizes the current progress in NSCL/P genetics research through a bibliometric analysis and comprehensively depicts the overall research landscape from multiple dimensions, including publication output, country, institution, author, discipline, journal, literature, and keywords. Research hotspots were categorized into four main research directions, revealing a clear research transition from genetic locus screening and candidate gene validation to mechanistic exploration of molecular pathways and integrated multi-omics analysis. The chronicle of keyword bursts further indicates that this transition is still ongoing, as the most recent bursts that remain active through 2026—rare variants, identify, and identification—signal that the discovery of rare, high-penetrance variants and their mechanistic characterization will remain the dominant direction of the field in the coming years. Further bioinformatic analysis identified the core signaling pathways and hub genes underlying NSCL/P pathogenesis induced by two typical environmental risk factors, alcohol and nicotine. Notably, TGF-β signaling and apoptosis were selectively enriched among alcohol-induced targets, whereas the HIF-1α/VEGF axis was preferentially implicated in nicotine-induced targets, highlighting both convergent and exposure-specific molecular foundations of gene–environment interplay. Investigations into gene–environment interactions and molecular mechanisms remain central research priorities. With the continuous advancement of sequencing technologies, single-cell sequencing and multi-omics integration have been increasingly applied to explore craniofacial development and its underlying molecular regulatory networks, and these approaches are expected to further dissect the molecular mechanisms of NSCL/P. The hub genes and enriched pathways identified in this study should be regarded as computationally prioritized hypotheses that warrant experimental validation in cellular and animal models. After further verification, these candidate targets may first inform practical preventive measures, such as genotype-guided management of periconceptional exposures, whereas pathway-based molecular interventions, although currently supported only by proof-of-concept evidence from animal models, may serve as a longer-term research direction. Relevant findings will further facilitate clinical translation and accelerate the prevention, diagnosis, and intervention of NSCL/P.

## Data Availability

The original contributions presented in the study are included in the article/Supplementary Material, further inquiries can be directed to the corresponding authors.
