# Supplementary material for: Global trends and hotspots of genetic research on non-syndromic cleft lip with or without cleft palate: a bibliometric and bioinformatics analysis
Source: Front Genet. 2026 Oct 12;17:1907333. doi: 10.3389/fgene.2026.1907333 (PMC13696031; doi:10.3389/fgene.2026.1907333)
Supplement: Supplementary file 1 [file Table1.docx]

Supplementary Table 1

| Number | Queries |
| --- | --- |
| #1 | TS = ("non-syndromic oral cleft*" OR "non-syndromic cleft lip*" OR "non-syndromic cleft palate*" OR "non-syndromic orofacial cleft*" OR "non-syndromic cleft lip with or without cleft palate" OR "non-syndromic cleft lip and palate" OR "non-syndromic CL/P" OR "non-syndromic CLP" OR "nonsyndromic oral cleft*" OR "nonsyndromic orofacial cleft*" OR "nonsyndromic cleft lip with or without cleft palate" OR "nonsyndromic cleft lip and palate" OR "nonsyndromic cleft lip" OR "nonsyndromic cleft palate" OR "nonsyndromic CL/P" OR "nonsyndromic CLP" OR "isolated oral cleft*" OR "isolated orofacial cleft*" OR "isolated cleft lip with or without cleft palate" OR "isolated cleft lip and palate" OR "isolated cleft lip*" OR "isolated cleft palate*" OR "isolated CL/P" OR "isolated CLP" OR "NSCL/P" OR "NSCLP") |
| #2 | TS = ("Genetic*" OR "Gene*" OR "Genomic" OR "Genotype" OR "Mutation*" OR "Variant*" OR "Allele" OR "Polymorphism*" OR "SNP" OR "Genome-wide association" OR "Sequencing" OR "WGS" OR "Whole Genome" OR "WES" OR "Whole Exome" OR "Next Generation Sequencing" OR "NGS" OR "CNV" OR "Copy Number Variant" OR "Deletion" OR "Duplication" OR "Hereditary" OR "Epigenetic*" OR "Methylation" OR "miRNA" OR "Expression" OR "Transcriptom*") |
| #3 | #1 AND #2 |
